# EXPLORING THE PERSPECTIVES OF NURSING STUDENTS ABOUT WORKING IN THE LONG-TERM CARE SECTOR

**DOI:** 10.1093/geroni/igae098.2796

**Published:** 2024-12-31

**Authors:** Winnie Sun, Janet McCabe, David Rudoler

**Affiliations:** Ontario Tech University, Lindsay, Ontario, Canada; Ontario Tech University, Oshawa, Ontario, Canada; Ontario Tech University, Oshawa, Ontario, Canada

## Abstract

**Background:**

The purpose of this study was to understand the perceptions of prospective registered nurses about working in the long-term care sector and identify workplace attributes that attract prospective nurses to the sector.

**Methods:**

A sequential mixed methods study was conducted with nursing students at Ontario Tech University. Focus groups (n=14) asked students to comment on views about working in the long-term care sector, and job attributes that may attract them to the sector. A cross-sectional survey asked students to respond to elicited choice job scenarios, informed by the thematic analysis of focus group interviews. The survey data (n=139) was analysed using least absolute deviations estimator. Willingness-to-pay (WTP) values of wages gained or forgone were generated for each job attribute.

**Results:**

Focus group interviews suggested fair compensation, optimal client-to-staff ratios, unionized work environments, comprehensive benefits packages, and flexible work arrangements were important job attributes. In survey results, 18.0% expressed interest in working in long-term care sector compared to 75.5% in acute care. Regression analysis suggested that higher amounts of paid vacation (WTP: -5.983; 95% CI: -13.749, -0.037) and higher risk of injury (WTP: 0.684; 95% CI: 0.124, 1.208) were associated with work in the long-term care sector.

**Conclusion:**

Long-term care can attract nurses by offering flexible options such as part-time positions and paid vacation, and by actions that potentially mitigate the risk of workplace injury, violence, and abuse. Nursing students should be shown the positive aspects of working with older adults and dispel negative perceptions about the long-term care sector.

